# Circulation of influenza A and B in the Czech Republic from 2000-2001 to 2015-2016

**DOI:** 10.1186/s12879-019-3783-z

**Published:** 2019-02-14

**Authors:** Martina Havlickova, Sophie Druelles, Helena Jirincova, Radomira Limberkova, Alexander Nagy, Anvar Rasuli, Jan Kyncl

**Affiliations:** 1 National Reference Laboratory for Influenza and Other Respiratory Viruses, National Institute of Public Health, Prague, Czech Republic; 2 Sanofi Pasteur, Lyon, France; 3 State Veterinary Institute, Prague, Czech Republic; 4 Department of Infectious Diseases Epidemiology, National Institute of Public Health, Prague, Czech Republic; 5 Department of Epidemiology and Biostatistics, 3rd Medical Faculty, Charles University, Prague, Czech Republic

**Keywords:** Czech Republic, Epidemiology, Influenza A, Influenza B, Influenza surveillance, Lineage

## Abstract

**Background:**

To improve national influenza vaccination recommendations, additional data on influenza A and B virus circulation are needed. Here, we describe the circulation of influenza A and B in the Czech Republic during 16 seasons.

**Methods:**

This was a retrospective analysis of data collected from the 2000–2001 to 2015–2016 influenza seasons by the Czech Republic national influenza surveillance network. Influenza was confirmed and viral isolates subtyped by virological assays followed by antigen detection or by reverse transcriptase-polymerase chain reaction.

**Results:**

Of 16,940 samples collected, 5144 (30.4%) were influenza-positive. Influenza A represented 78.6% of positive cases overall and accounted for more than 55.0% of all influenza cases in every season, except for 2005–2006 (6.0%). Both A/H1N1 and A/H3N2 were detected in most seasons, except for 2001–2002 and 2003–2004 (only A/H3N2), and 2007–2008 and 2009–2010 (only A/H1N1). Influenza B represented 21.4% of positive cases overall (range, 0.0–94.0% per season). Both influenza B lineages were detected in three seasons, a single B lineage in 11, and no B strain in two. For the 11 seasons where influenza B accounted for ≥20% of positive cases, the dominant lineage was Yamagata in six and Victoria in four. In the remaining season, the two lineages co-circulated. For two seasons (2005–2006 and 2007–2008), the B lineage in the trivalent influenza vaccine did not match the dominant circulating B lineage.

**Conclusions:**

In the Czech Republic, during the 2000–2001 to 2015–2016 influenza seasons, influenza virus circulation varied considerably. Although influenza A accounted for the most cases in almost all seasons, influenza B made a substantial, sometimes dominant, contribution to influenza disease.

## Background

Consistent with European Union Council recommendations [1], most European member states have national policies recommending seasonal influenza vaccination for all people at risk of influenza complications. In the Czech Republic, recommendations for seasonal influenza vaccination have been available since 2011 [2]. Reflecting the recommendations of the World Health Organization (WHO), targeted groups include individuals with chronic illnesses and their caregivers, pregnant women, healthcare workers, and adults aged 65 years and older [3].

Despite these recommendations, influenza vaccination coverage in the Czech Republic is substantially below the WHO, European, and Czech targets of 75% for older adults and individuals with chronic illnesses [1, 2, 4]. Although vaccination coverage is not routinely measured in the Czech Republic [5], recent analyses have reported a vaccination coverage rate of ~ 25% for older adults and persons with underlying comorbidities in the Czech Republic [6], and moreover, that vaccination coverage could be decreasing [7].

Most influenza vaccines in the Czech Republic are trivalent, containing two strains of influenza A (H1N1 and H3N2) and a single B strain lineage [8]. However, since the early 2000s, two genetically distinct lineages of influenza B virus, Yamagata and Victoria, have co-circulated worldwide, which can result in mismatches between the vaccine and the predominant circulating B strain [8]. Quadrivalent influenza vaccines including both B strain lineages have been developed to prevent these B lineage mismatches [8–11], and have been available in the Czech Republic since the 2017–2018 influenza season.

To control influenza spread and encourage seasonal influenza vaccination, national epidemiologic data are needed to help develop policies. Since the 1980s, the Czech Republic has had a relatively stable population of approximately 10.5 million [12]. Surveillance data from the Czech Republic collected between 1999 and 2013 indicated that influenza was responsible for approximately 1408 deaths each year, 1.31% of all-cause mortality, and 1.86% of circulatory disease-related mortality [13]. Limited data are available on the burden of influenza in elderly adults in the Czech Republic [14], but data have not been published on the burden overall or on the distribution of influenza A and B strains. Here, we describe the results of a retrospective analysis of influenza A and B virus circulation in the Czech Republic from 2000–2001 to 2015–2016.

## Methods

### Study design

This was a retrospective analysis of data collected by the national influenza surveillance network of the Czech Republic. The primary objective was to describe circulation of influenza A and B viruses during the 16 consecutive seasons from 2000–2001 to 2015–2016. Ethical approval was not required for this study.

### Surveillance of acute respiratory infection (ARI)

In the Czech Republic, data on influenza and other ARIs have been collected weekly throughout the year since 1968. The data are collected by more than 2000 general practitioners and more than 1000 pediatricians as part of a sentinel system that covers all 14 regions and approximately 50% of the population in the Czech Republic [15]. Until 2003, data were collected from individuals meeting the WHO case definition of ARI [16]. Starting in 2004, data were also included from individuals meeting the European Centre for Disease Control and Prevention case definition of influenza-like illness (ILI) [17]. Additionally, the Ministry of Health of the Czech Republic manages a surveillance system in which sentinel physicians in each of the 14 regions (usually two physicians per region) collect swabs from their ILI/ARI patients between week 40 of each year and week 20 of the following year.

In addition to this sentinel surveillance, since 2009, non-sentinel data and samples have been routinely collected from all hospitalized individuals in the Czech Republic with severe ARI [16]. Data were categorized by age group: 0–5 years (preschoolers), 6–14 years (age range of compulsory education in the Czech Republic), 15–24 years (i.e., until the age when most regular students finish their university study), 25–59 years, and ≥ 60 years (usual retirement age in the Czech Republic).

### Laboratory methods

Nasopharyngeal swabs were collected according to the WHO Manual for the Laboratory Diagnosis and Virological Surveillance of Influenza [18] and were sent to the Czech Republic National Reference Laboratory to confirm influenza virus infection and identify the influenza type or subtype.

For samples collected before 2009, influenza infection was confirmed by virological assays and virus was typed/subtyped by antigen detection. Briefly, influenza viruses were proliferated in the allantoic sac of chicken embryos or in Madin-Darby canine kidney cells, and antigens were detected using a Directigen™ EZ Flu A + B kit (Becton-Dickenson, San Jose, CA, USA) or a QuickVue Influenza A + B test (Quidel, San Diego, CA, USA).

For samples collected during or after 2009, influenza infection was confirmed and typed/subtyped by reverse transcription-polymerase chain reaction. Briefly, nucleic acids were extracted using MagNAPure LC kits and a MagNAPure Compact instrument (Roche Molecular Systems, Pleasanton, CA, USA) or a RTP DNA/RNA Virus Mini Kit (Stratec Biomedical Systems, Birkenfeld, Germany). For most samples, influenza A and B were detected using the WHO method with primers and probes obtained from the US Centers for Disease Control and Prevention (Atlanta, GA, USA), an AgPath-ID One step RT PCR kit (Thermo Fisher Scientific, Waltham, MA, USA), the MeltMan reverse transcriptase-quantitative polymerase chain reaction system [19], and SYTO® 82 Orange Fluorescent Nucleic Acid Stain (Thermo Fisher Scientific). In some cases, influenza B and B lineages were detected according to Daum et al. (2007) [20] using primers and probes described by Schweiger et al. [21] and Watzinger et al. [22]. Hemagglutination inhibition assays may also have been used to detect the B lineage. Data on specific A/H1N1 and A/H3N2 strains and drift variants were not available.

### Statistical analysis

Influenza seasons were defined as week 40 of a given year to week 20 of the following year. The peak of influenza activity was defined as the epidemiological week with the highest number of ARI cases during a given season. As described previously [23], seasons were considered to have significant circulation of influenza A or influenza B when either represented at least 20% of all influenza cases reported. The dominant circulating viruses for each season were defined as the A strain identified in ≥70% of influenza A-positive samples and the B lineage identified in ≥70% of influenza B-positive samples. A season was considered to have a B-strain mismatch if the B lineage in the vaccine was different from the dominant circulating lineage. A season was considered to have a partial mismatch when the two B lineages co-circulated in approximately equal proportions. All data were analyzed using Excel (Microsoft, Redmond, WA). Missing data were not replaced, and only descriptive statistics were calculated.

## Results

### Samples and confirmed influenza cases

During the 16 seasons from 2000–2001 to 2015–2016, 16,940 samples were collected and tested (Table 1). The fewest samples (315) were collected in 2003–2004 and, excluding the 2009–2010 A(H1N1) pandemic season, the most (2425) were collected in 2012–2013. During the 2009–2010 pandemic season, 3864 samples were collected.

**Table 1:** Samples processed and positive for influenza by season

Season	Source^a^	Samples processed	Influenza-positive		Influenza A cases		Influenza B cases	
			*n*	%	*n*	%^b^	*n*	%^b^
2000–2001	Total	509	59	11.6	50	84.7	9	15.3
2001–2002	Total	536	24	4.5	19	79.2	5	20.8
2002–2003	Total	393	97	24.7	55	56.7	42	43.3
2003–2004	Total	315	41	13.0	40	97.6	1	2.4
2004–2005	Total	827	155	18.7	115	74.2	40	25.8
2005–2006	Total	731	134	18.3	8	6.0	126	94.0
2006–2007	Total	723	118	16.3	118	100.0	0	0.0
2007–2008	Total	729	197	27.0	135	68.5	62	31.5
2008–2009	Total	781	205	26.2	157	76.6	48	23.4
2009–2010	Sentinel	676	211	31.2	210	99.5	1	0.5
	Non-sentinel	3188	1064	33.4	1064	100.0	0	0.0
	Total	3864	1275	33.0	1274	99.9	1	0.1
2010–2011	Sentinel	535	211	39.4	140	66.4	71	33.6
	Non-sentinel	590	233	39.5	205	88.0	28	12.0
	Total	1125	444	39.5	345	77.7	99	22.3
2011–2012	Sentinel	578	151	26.1	117	77.5	34	22.5
	Non-sentinel	365	102	27.9	84	82.4	18	17.6
	Total	943	253	26.8	201	79.4	52	20.6
2012–2013	Sentinel	543	241	44.4	163	67.6	78	32.4
	Non-sentinel	1882	938	49.8	737	78.6	201	21.4
	Total	2425	1179	48.6	900	76.3	279	23.7
2013–2014	Sentinel	415	22	5.3	19	86.4	3	13.6
	Non-sentinel	203	11	5.4	11	100.0	0	0.0
	Total	618	33	5.3	30	90.9	3	9.1
2014–2015	Sentinel	471	216	45.9	126	58.3	90	41.7
	Non-sentinel	651	308	47.3	213	69.2	95	30.8
	Total	1122	524	46.7	339	64.7	185	35.3
2015–2016	Sentinel	492	139	28.3	70	50.4	69	49.6
	Non-sentinel	807	267	33.1	188	70.4	79	29.6
	Total	1299	406	31.3	258	63.5	148	36.5
All seasons	Total	16,940	5144	30.4	4044	78.6	1100	21.4

^a^Until 2009, sentinel and non-sentinel samples were reported together

^b^Proportion of influenza-positive cases

Of the 16,940 samples, 5144 (30.4%) tested positive for influenza. The proportion of influenza-positive cases was lowest (4.5%; *n* = 24) during the 2001–2002 season and, excluding the 2009–2010 pandemic season, was highest (48.6%; *n* = 1179) during the 2012–2013 season. During the pandemic season, 1275 samples (33.0%) tested positive for influenza.

### Temporal characteristics of ARI cases

**Fig. 1:**
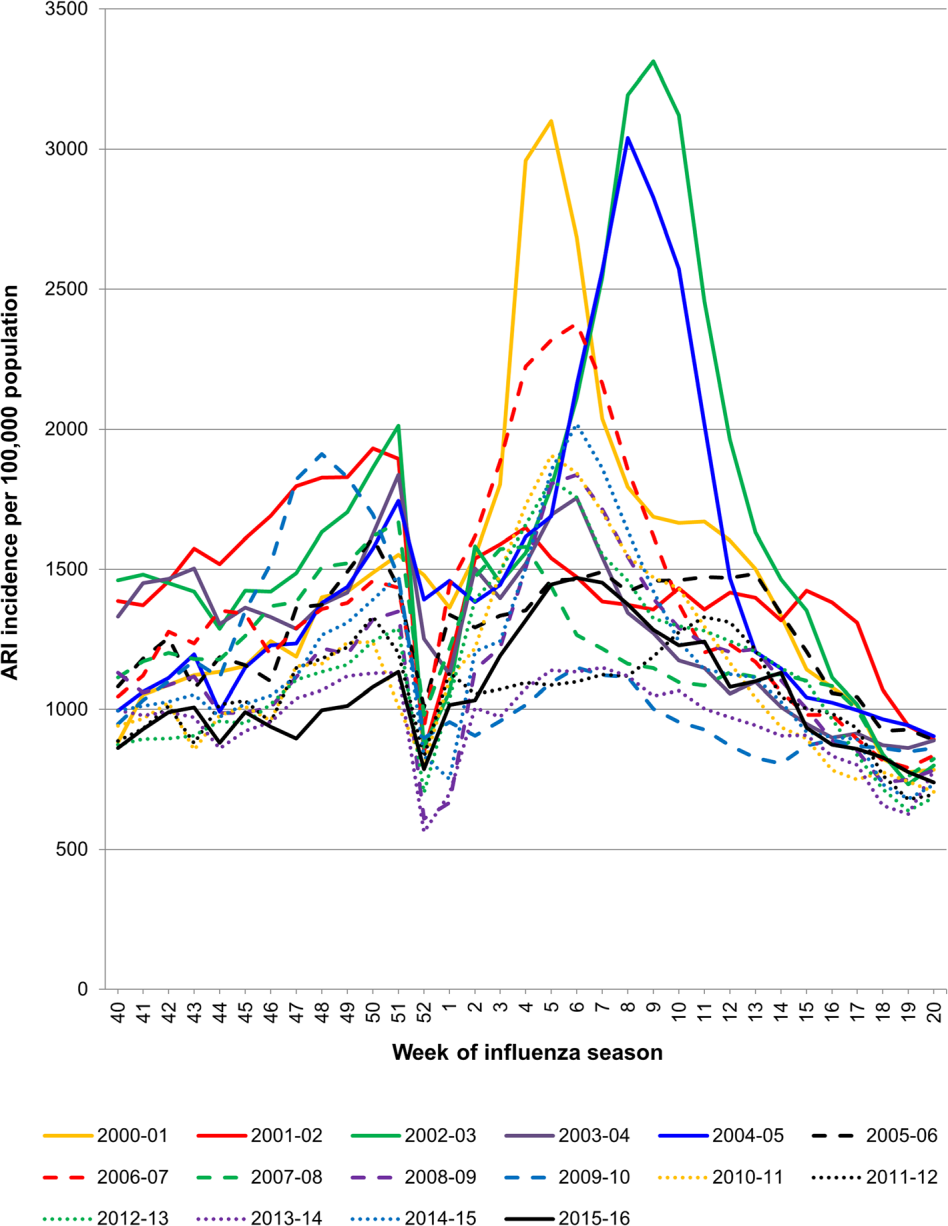
Incidence of acute respiratory infection (ARI) cases in the Czech Republic by season and epidemiological week

The earliest peak of ARI cases (week 48) occurred during the 2009–2010 pandemic season and the latest (week 11) during the 2011–2012 season (Fig. 1). Excluding the 2009–2010 pandemic season, ARI cases followed two main patterns: a continuous low or moderate number (seen in the 2001–2002, 2003–2004, 2005–2006, 2007–2008, 2011–2012, 2013–2014, and 2015–2016 seasons); or a concentration of cases over 10 to 15 weeks with a prominent peak (seen in the 2000–2001, 2002–2003, 2004–2005, 2006–2007, 2008–2009, 2010–2011, 2012–2013, and 2014–2015 seasons). For most of the seasons, ARI incidence decreased at week 52, coinciding with the Czech Republic’s winter vacations.

### Circulation of influenza A and B

**Fig. 2:**
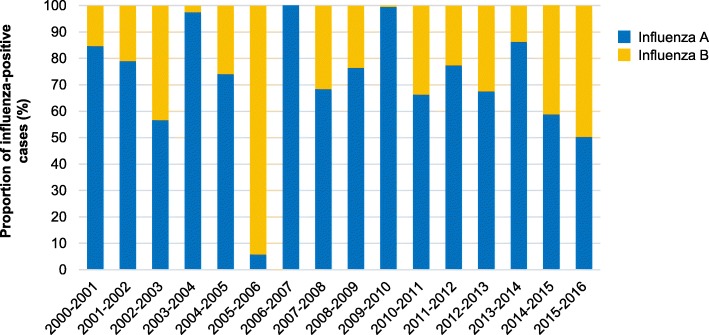
Proportion of cases positive for influenza A and B by season

Influenza A was responsible for 78.6% of confirmed influenza cases overall, and accounted for more than 55% of all influenza cases in every season, except for 2005–2006 when it was identified in just 6% (8/134) of influenza-positive samples (Table 1). Influenza B accounted for at least 20% of all influenza cases in 11 of the 16 seasons between 2000–2001 and 2015–2016, and represented 21.4% (1100/5144) of confirmed influenza cases overall. The highest proportion of samples positive for influenza B was during the 2005–2006 season (94%; 126/134), and the lowest during the subsequent 2006–2007 season (0%; 0/118) (Table 1 and Fig. 2). The proportion of influenza B was also very low during the 2003–2004 season (2.4%; 1/41) and the 2009–2010 pandemic season (0.1%; 1/1275).

For seasons where data from sentinel and non-sentinel sources were tabulated separately (2009–2010 to 2015–2016), overall rates of influenza positivity were similar (Table 1). However, the proportion of influenza A was consistently lower for sentinel than non-sentinel samples, whereas the proportion of influenza B was lower for non-sentinel samples. For example, during the 2010–2011 season, influenza A accounted for 66% (140/211) of positive samples from sentinel sources but 88% (205/233) of those from non-sentinel sources; and influenza B accounted for 34% (71/211) of positive samples from sentinel sources but only 12% (28/233) from non-sentinel sources.

The proportions of influenza A cases in different age groups were similar between the 2010–2011 and 2015–2016 seasons with most cases found in individuals aged 25 years or older (Table 2). In contrast, during the 2009–2010 pandemic season, more influenza A cases were seen in 15–24-year-olds (23.7% vs. 3.5–10.4% seen between 2010–2011 and 2015–2016) and fewer in adults older than 60 years (9.3% vs. 13.3–60.2%). Most influenza B cases were found in 6–14-year-olds in 2010–2011 (41.4%; 41/99) and 2011–2012 (36.5%; 19/52) but were more represented in the adult age groups from 2012–2013 to 2015–2016.

**Table 2:** Samples positive for influenza in different age groups

Season^a^	Category	Influenza A, *n* (%)			Influenza B, *n* (%)		
		Sentinel	Non-sentinel	Total	Sentinel	Non-sentinel	Total
2009–2010	Samples processed	210	1056^b^	1268	1	0	1
	Age group, y						
	0–5 y	27 (12.9)	41 (3.9)	68 (5.4)	1 (100.0)	0 (0.0)	1 (100.0)
	6–14 y	74 (35.2)	91 (8.6)	165 (13.0)	0 (0.0)	0 (0.0)	0 (0.0)
	15–24 y	60 (28.6)	240 (22.7)	300 (23.7)	0 (0.0)	0 (0.0)	0 (0.0)
	25–59 y	43 (20.5)	572 (54.1)	615 (48.5)	0 (0.0)	0 (0.0)	0 (0.0)
	≥ 60 y	6 (2.9)	112 (10.6)	118 (9.3)	0 (0.0)	0 (0.0)	0 (0.0)
2010–2011	Samples processed	140	205	345	71	28	99
	Age group, *n* (%)						
	0–5 y	14 (10.0)	7 (3.4)	21 (6.1)	2 (2.8)	1 (3.6)	3 (3.0)
	6–14 y	44 (31.4)	3 (1.5)	47 (13.6)	37 (52.1)	4 (14.3)	41 (41.4)
	15–24 y	24 (17.1)	12 (5.9)	36 (10.4)	19 (26.8)	3 (10.7)	22 (22.2)
	25–59 y	50 (35.7)	115 (56.1)	165 (47.8)	10 (14.1)	10 (35.7)	20 (20.2)
	≥ 60 y	8 (5.7)	68 (33.2)	76 (22.0)	3 (4.2)	10 (35.7)	13 (13.1)
2011–2012	Samples processed	117	84	201	34	18	52
	Age group, *n* (%)						
	0–5 y	22 (18.8)	3 (3.6)	25 (12.4)	2 (5.9)	1 (5.6)	3 (5.8)
	6–14 y	35 (29.9)	3 (3.6)	38 (18.9)	17 (50.0)	2 (11.1)	19 (36.5)
	15–24 y	15 (12.8)	3 (3.6)	18 (9.0)	5 (14.7)	5 (27.8)	10 (19.2)
	25–59 y	37 (31.6)	35 (41.7)	72 (35.8)	8 (23.5)	7 (38.9)	15 (28.8)
	≥ 60 y	8 (6.8)	40 (47.6)	48 (23.9)	2 (5.9)	3 (16.7)	5 (9.6)
2012–2013	Samples processed	162^c^	737	899	77^c^	201	278
	Age group, *n* (%)						
	0–5 y	14 (8.6)	20 (2.7)	34 (3.8)	6 (7.8)	6 (3.0)	12 (4.3)
	6–14 y	35 (21.6)	8 (1.1)	43 (4.8)	36 (46.8)	10 (5.0)	46 (16.5)
	15–24 y	19 (11.7)	16 (2.2)	35 (3.9)	11 (14.3)	9 (4.5)	20 (7.2)
	25–59 y	73 (45.1)	371 (50.3)	444 (49.4)	18 (23.4)	93 (46.3)	111 (39.9)
	≥ 60 y	21 (13.0)	322 (43.7)	343 (38.2)	6 (7.8)	83 (41.3)	89 (32.0)
2013–2014	Samples processed	19	11	30	3	0	3
	Age group, *n* (%)						
	0–5 y	2 (10.5)	1 (9.1)	3 (10.0)	0 (0.0)	0 (0.0)	0 (0.0)
	6–14 y	4 (21.1)	0 (0.0)	4 (13.3)	2 (66.7)	0 (0.0)	2 (66.7)
	15–24 y	1 (5.3)	2 (18.2)	3 (10.0)	0 (0.0)	0 (0.0)	0 (0.0)
	25–59 y	11 (57.9)	5 (45.5)	16 (53.3)	1 (33.3)	0 (0.0)	1 (33.3)
	≥ 60 y	1 (5.3)	3 (27.3)	4 (13.3)	0 (0.0)	0 (0.0)	0 (0.0)
2014–2015	Samples processed	126	213	339	89^c^	95	184
	Age group, *n* (%)						
	0–5 y	21 (16.7)	0 (0.0)	21 (6.2)	4 (4.5)	0 (0.0)	4 (2.2)
	6–14 y	23 (18.3)	0 (0.0)	23 (6.8)	2 (2.2)	0 (0.0)	2 (1.1)
	15–24 y	26 (20.6)	6 (2.8)	32 (9.4)	2 (2.2)	1 (1.1)	3 (1.6)
	25–59 y	33 (26.2)	26 (12.2)	59 (17.4)	80 (89.9)	14 (14.7)	94 (51.1)
	≥ 60 y	23 (18.2)	181 (85.0)	204 (60.2)	1 (1.1)	80 (84.2)	81 (44.0)
2015–2016	Samples processed	70	188	258	69	79	148
	Age group, n (%)						
	0–5 y	12 (17.1)	2 (1.1)	14 (5.4)	8 (11.6)	0 (0.0)	8 (5.4)
	6–14 y	19 (27.1)	2 (1.1)	21 (8.1)	35 (50.7)	0 (0.0)	35 (23.6)
	15–24 y	5 (7.1)	4 (2.1)	9 (3.5)	17 (24.6)	5 (6.3)	22 (14.9)
	25–59 y	28 (40.0)	101 (53.7)	129 (50.0)	9 (13.0)	42 (53.2)	51 (34.5)
	≥ 60 y	6 (8.6)	79 (42.0)	85 (32.9)	0 (0.0)	32 (40.5)	32 (21.6)

^a^Data per age group were not available for the seasons before 2009–2010 due to differences in data reporting systems

^b^Excludes 8 samples that had no age information

^c^Excludes 1 sample that had no age information

### Circulation of influenza A/H1N1 and A/H3N2

The strain of influenza A (A/H1N1 or A/H3N2) was identified for 3246 (80.3%) of the 4044 influenza A cases (Table 3). Overall, 2168 cases (66.8%) were caused by A/H1N1, although more than one third of these (39.3%; *n* = 853) were from the 2009–2010 pandemic season. Both A strains were identified from the samples characterized in most seasons except for 2001–2002 and 2003–2004 where only A/H3N2 was identified, and 2007–2008 and 2009–2010 where only A/H1N1 was identified. Of the 15 seasons with significant A virus circulation (≥ 20% of influenza cases; see Table 1), A/H1N1 was the dominant circulating strain in six seasons and A/H3N2 in seven. In the remaining two seasons (2004–2005 and 2013–2014), the two A strains co-circulated in nearly equal proportions.

**Table 3:** A/H1N1 and A/H3N2 cases detected during each influenza season

Season	Total A samples characterized	A/H1N1		A/H3N2		Dominant strain
		*n*	%	*n*	%	
2000–2001	48	44	91.7	4	8.3	H1N1
2001–2002	13	0	0.0	13	100.0	H3N2
2002–2003	53	5	9.4	48	90.6	H3N2
2003–2004	33	0	0.0	33	100.0	H3N2
2004–2005	76	33	43.4	43	56.6	Co-circulation
2005–2006	7	1	14.3	6	85.7	H3N2
2006–2007	109	1	0.9	108	99.1	H3N2
2007–2008	130	130	100.0	0	0.0	H1N1
2008–2009	139	6	4.3	133	95.7	H3N2
2009–2010	853	853	100.0	0	0.0	H1N1
2010–2011	233	228	97.6	5	2.2	H1N1
2011–2012	153	13	8.5	140	91.5	H3N2
2012–2013	814	573	70.4	241	29.6	H1N1
2013–2014	25	9	36.0	16	64.0	Co-circulation
2014–2015	326	50	15.3	276	84.7	H3N2
2015–2016	234	222	94.9	12	5.1	H1N1
Total	3246	2168^a^	66.8	1078	33.2	H1N1^a^

^a^Not adjusted for the high number of cases from the 2009–2010 pandemic

### Circulation of influenza B lineages and occurrence of mismatches with the vaccine B strain

The lineage was identified for 649 (59.0%) of the 1100 influenza B cases (Table 4). Of these, B Yamagata accounted for 341 cases (52.5%), B Victoria for 305 cases (47.0%), and both lineages for 3 cases (0.5%). Of the 16 seasons included in this study, both B lineages were detected in three, a single B lineage was detected in nine, and no influenza B was detected in four. Of the 11 seasons with significant B virus circulation, the dominant lineage was Yamagata in six and Victoria in four. In the remaining season (2015–2016), the two B lineages co-circulated in nearly equal proportions. The B strain in the trivalent influenza vaccine did not match the dominant circulating B strain in two of these 11 seasons (2005–2006 and 2007–2008). The 2015–2016 vaccine was considered to have had a partial mismatch with the circulating B lineages because influenza viruses of both lineages co-circulated.

**Table 4:** B lineage detected during each influenza season and comparison with the B lineage recommended by the World Health Organization for the trivalent influenza vaccine

Season	Total B samples characterized	Yamagata lineage		Victoria lineage		Both lineages		Dominant circulating B lineage	B lineage in the trivalent vaccine	Mismatch
		*n*	%	*n*	%	*n*	%			
2000–2001	9	9	100.0	0	0.0	0	0.0	Yamagata	Yamagata	No
2001–2002	5	5	100.0	0	0.0	0	0.0	Yamagata	Yamagata	No
2002–2003	38	0	0.0	38	100.0	0	0.0	Victoria	Victoria	No
2003–2004	0	–	–	–	–	–	–	Unknown	Victoria	Unknown
2004–2005	40	40	100.0	0	0.0	0	0.0	Yamagata	Yamagata	No
2005–2006	122	0	0.0	122	100.0	0	0.0	Victoria	Yamagata	Yes
2006–2007	0	–	–	–	–	–	–	Unknown	Victoria	Unknown
2007–2008	62	62	100.0	0	0.0	0	0.0	Yamagata	Victoria	Yes
2008–2009	12	10	83.3	2	16.7	0	0.0	Yamagata	Yamagata	No
2009–2010	0	–	–	–	–	–	–	Unknown	Victoria	Unknown
2010–2011	30	0	0.0	30	100.0	0	0.0	Victoria	Victoria	No
2011–2012	52	4	7.7	48	92.3	0	0.0	Victoria	Victoria	No
2012–2013	117	117	100.0	0	0.0	0	0.0	Yamagata	Yamagata	No
2013–2014	0	–	–	–	–	–	–	Unknown	Yamagata	No
2014–2015	40	40	100.0	0	0.0	0	0.0	Yamagata	Yamagata	No
2015–2016	122	54	44.3	65	53.3	3	2.5	Co-circulation	Yamagata	Partial
Total	649	341	52.5	305	47.0	3	0.5	–	–	–

## Discussion

In this report, we describe the circulation of influenza viruses in the Czech Republic based on data collected from the national influenza surveillance network from 2000–2001 to 2015–2016. Although influenza A accounted for most influenza cases in almost all of the seasons, they typically co-circulated with influenza B viruses. Influenza B represented just over one-fifth (21.4%) of all confirmed influenza cases, although the proportion in each season ranged from as low as 0% (2006–2007) to as high as 94% (2005–2006). This is in line with European epidemiological data collected between 2001–2002 and 2010–2011, where influenza B represented 23% of influenza cases overall and from 1 to 60% of the cases each year [8]. Influenza B frequency is known to vary widely between seasons in many countries worldwide [24].

The results presented here are among the first describing the circulation of influenza strains in the Czech Republic. Some data for the Czech Republic were recently published by the Global Influenza Hospital Surveillance Network (GIHSN) for the 2014–2015 influenza season [25]. Although the GIHSN collects data on only severe cases, it found that influenza B accounted for about one-third of influenza cases during 2014–2015.

In most seasons with B strain circulation, a single lineage dominated, and generally these dominant lineages matched those reported during the same seasons in several other European countries [23, 26, 27]. In all but two of the 16 seasons (2005–2006 and 2007–2008), the dominant circulating B lineage was the same as the B lineage in the seasonal influenza vaccine. In one season (2015–2016), the two B lineages co-circulated in nearly equal proportions, resulting in a partial mismatch with the vaccine B lineage. Similarly, data from the US and Europe show that major mismatches occurred in 2005–2006, 2007–2008, and 2008–2009 [11, 26, 27]. Such mismatches between the B lineage in trivalent vaccines and the dominant circulating B lineage represent a missed opportunity to protect against influenza. Quadrivalent influenza vaccines containing both B lineages have been developed to avoid this [8–11] and are now included in WHO guidelines [28]. These quadrivalent influenza vaccines are expected to further reduce the burden and economic impact of influenza beyond that provided by trivalent vaccines [29, 30].

Analysis of influenza cases by week revealed that some years had a clear peak of influenza cases but that others had a more even distribution and a weaker peak. How each influenza strain contributed to these patterns was not analyzed, although the circulation of influenza subtypes and lineages often changes over the course of a single season [25, 31, 32]. This complicates predicting which strains and B lineages will dominate each year and further emphasizes the need for quadrivalent vaccines.

Interestingly, for seasons where data from sentinel and non-sentinel sources were tabulated separately (2009–2010 to 2015–2016), overall rates of influenza positivity were similar but the proportion of influenza A was consistently higher for non-sentinel than sentinel samples, with the reverse true for influenza B. As the non-sentinel samples were collected only from hospitalized individuals, this suggests that influenza A led to severe ARI more often than influenza B.

This report provides essential information about the circulation of influenza viruses, but low numbers limited the analyses that could be performed. For example, we could not effectively stratify by risk category or influenza severity, although the oldest and youngest individuals, pregnant women, and individuals with chronic conditions are well known to be at increased risk for severe influenza [25, 31–33]. In addition, very few influenza cases were detected in some seasons, increasing the uncertainty around the circulation of individual A strains and B lineages. For most of the seasons, ARI incidence also decreased around the Czech winter vacation season, which is likely due to reduced movement and mixing of the population and perhaps also due to reduced ARI reporting, since many healthcare personnel also take vacation around this time. Regardless, the results highlight the variability of influenza strain circulation and confirm the substantial risk for mismatches between the B lineage in trivalent vaccines and the dominant circulating B lineage.

Another potential limitation of the current analysis was heterogeneity in methods across seasons and between sentinel and non-sentinel sources. Case ascertainment by the surveillance system in the Czech Republic was based on the WHO definition of ARI for 2001–2003, but starting in 2004, it also included the European Centre for Disease Control and Prevention definition of ILI. Also, prior to 2009, influenza was confirmed by virological tests and typed/subtyped by antigen assays, but thereafter, influenza was confirmed and typed by reverse transcription-polymerase chain reaction. This did not appear to have influenced the overall positivity rate, although the frequency of influenza differed. However, this should not affect the conclusions about B lineage mismatches because a single B lineage strain was clearly dominant in most seasons.

## Conclusions

Surveillance data from the Czech Republic showed that influenza virus circulation varied considerably during the 16 seasons from 2000–2001 to 2015–2016. Influenza A/H1N1 or A/H3N2 strains accounted for most cases in almost all seasons, and were responsible for more than three-quarters of cases overall. Influenza B made a substantial, sometimes dominant, contribution, yet the B-strain lineage in seasonal trivalent vaccines did not always match the dominant circulating B-strain lineage. The results from this study should help further inform influenza vaccination policy in the Czech Republic.

## Abbreviations

ARI: Acute respiratory infection
GIHSN: Global Influenza Hospital Surveillance Network
ILI: Influenza-like illness
WHO: World Health Organization

## References

[1] Council of the European Union (2009). Council Recommendation of 22 December 2009 on seasonal influenza vaccination. Off J Eur. Union.

[2] Recommended procedure for vaccination against seasonal influenza in the Czech Republic [article in Czech]. Ministry of Health of the Czech Republic; 2011. http://www.mzcr.cz/dokumenty/doporuceny-postup-pro-ockovani-proti-sezonni-chripce_5194_1985_5.html. Accessed 23 Jan 2017.

[3] European Centre for Disease Prevention and Control. Seasonal influenza vaccination in Europe. Overview of vaccination recommendations and coverage rates in the EU Member States for the 2012–13 influenza season. ECDC; 2015. https://ecdc.europa.eu/sites/portal/files/media/en/publications/Publications/Seasonal-influenza-vaccination-Europe-2012-13.pdf. Accessed 28 Sept 2016.

[4] Prevention and control of influenza pandemics and annual epidemics. Resolution WHA56.19 of 28 May 2003. Geneva: World Health Organization; 2003. http://www.who.int/immunization/sage/1_WHA56_19_Prevention_and_control_of_influenza_pandemics.pdf. Accessed 9 Aug 2017.

[5] Seasonal influenza vaccination programme, country profile: Czech Republic, 2012–2013 Season. European Centre for Disease Control and Prevention, Stockholm, Sweden. https://ecdc.europa.eu/sites/portal/files/media/en/publications/Report%20Assets/seasonal-vaccination-coverage-in-europe-2012-13/Seasonal-Influenza-Vaccination-Programme-Country-Profile-Czech-Republic.pdf (2015). Accessed 16 Jun 2016.

[6] European Centre for Disease Prevention and Control. Seasonal influenza vaccination in Europe. Vaccination recommendations and coverage rates in the EU Member States for eight influenza seasons: 2007–2008 to 2014–2015. Stockholm: ECDC; 2017.

[7] Palache A, Oriol-Mathieu V, Fino M, Xydia-Charmanta M (2015). Seasonal influenza vaccine dose distribution in 195 countries (2004-2013): little progress in estimated global vaccination coverage. Vaccine.

[8] Ambrose CS, Levin MJ (2012). The rationale for quadrivalent influenza vaccines. Hum Vaccin Immunother.

[9] Belshe RB (2010). The need for quadrivalent vaccine against seasonal influenza. Vaccine.

[10] Hannoun C (2013). The evolving history of influenza viruses and influenza vaccines. Expert Rev Vaccines.

[11] Tisa V, Barberis I, Faccio V, Paganino C, Trucchi C, Martini M (2016). Quadrivalent influenza vaccine: a new opportunity to reduce the influenza burden. J Prev Med Hyg.

[12] Czech Republic Population Worldometers. http://www.worldometers.info/world-population/czech-republic-population/ (2018). Accessed 7 Sep 2018.

[13] Kyncl J, Havlickova M, Jirincova H. Influenza attributable mortality in the Czech Republic. Poster P-488. In: Options IX for the Control of Influenza, 24–28 August 2016. Chicago: ISIRV; 2016.

[14] Kovacs G, Kalo Z, Jahnz-Rozyk K, Kyncl J, Csohan A, Pistol A (2014). Medical and economic burden of influenza in the elderly population in central and eastern European countries. Hum Vaccin Immunother.

[15] World Health Organization. Influenza surveillance country profile - Czech Republic. WHO. http://www.euro.who.int/__data/assets/pdf_file/0003/272505/Country_profile_influenza_Czech-Republic_final_ENG_new-layout.pdf?ua=1 (2015). Accessed 10 Aug 2017.

[16] World Health Organization. WHO Regional Office for Europe guidance for sentinel influenza surveillance in humans WHO Regional Office for Europe. http://www.euro.who.int/__data/assets/pdf_file/0020/90443/E92738.pdf (2011). Accessed 9 Aug 2017.

[17] European Commission. Commission implementing decision of 8 August 2012 amending decision 2002/253/EC laying down case definitions for reporting communicable diseases to the community network under decision no 2119/98/EC of the European Parliament and of the council. Official J Eur Union. 2012;L262:1–56.

[18] World Health Organization Global Influenza Surveillance Network. Manual for the laboratory diagnosis and virological surveillance of influenza. WHO. http://apps.who.int/iris/bitstream/10665/44518/1/9789241548090_eng.pdf (2011). Accessed 15 Sep 2017.

[19] Nagy A, Cernikova L, Vitaskova E, Krivda V, Dan A, Dirbakova Z (2016). MeltMan: optimization, evaluation, and universal application of a qPCR system integrating the TaqMan qPCR and melting analysis into a single assay. PLoS One.

[20] Daum LT, Canas LC, Arulanandam BP, Niemeyer D, Valdes JJ, Chambers JP (2007). Real-time RT-PCR assays for type and subtype detection of influenza a and B viruses. Influenza Other Respir Viruses.

[21] Schweiger B, Zadow I, Heckler R, Timm H, Pauli G (2000). Application of a fluorogenic PCR assay for typing and subtyping of influenza viruses in respiratory samples. J Clin Microbiol.

[22] Watzinger F, Suda M, Preuner S, Baumgartinger R, Ebner K, Baskova L (2004). Real-time quantitative PCR assays for detection and monitoring of pathogenic human viruses in immunosuppressed pediatric patients. J Clin Microbiol.

[23] Caini S, Huang QS, Ciblak MA, Kusznierz G, Owen R, Wangchuk S (2015). Epidemiological and virological characteristics of influenza B: results of the global influenza B study. Influenza Other Respir Viruses.

[24] Paul Glezen W, Schmier JK, Kuehn CM, Ryan KJ, Oxford J (2013). The burden of influenza B: a structured literature review. Am J Public Health.

[25] Puig-Barbera J, Burtseva E, Yu H, Cowling BJ, Badur S, Kyncl J (2016). Influenza epidemiology and influenza vaccine effectiveness during the 2014-2015 season: annual report from the global influenza hospital surveillance network. BMC Public Health.

[26] Mosnier A, Caini S, Daviaud I, Bensoussan JL, Stoll-Keller F, Bui TT (2015). Ten influenza seasons in France: distribution and timing of influenza a and B circulation, 2003-2013. BMC Infect Dis.

[27] Heikkinen T, Ikonen N, Ziegler T (2014). Impact of influenza B lineage-level mismatch between trivalent seasonal influenza vaccines and circulating viruses, 1999-2012. Clin Infect Dis.

[28] World Health Organization (2012). Vaccines against influenza WHO position paper - November 2012. Wkly Epidemiol Rec.

[29] de Boer PT, van Maanen BM, Damm O, Ultsch B, Dolk FCK, Crepey P (2017). A systematic review of the health economic consequences of quadrivalent influenza vaccination. Expert Rev Pharmacoecon Outcomes Res.

[30] Uhart M, Bricout H, Clay E, Largeron N (2016). Public health and economic impact of seasonal influenza vaccination with quadrivalent influenza vaccines compared to trivalent influenza vaccines in Europe. Hum Vaccin Immunother..

[31] Puig-Barbera J, Natividad-Sancho A, Trushakova S, Sominina A, Pisareva M, Ciblak MA (2016). Epidemiology of hospital admissions with influenza during the 2013/2014 northern hemisphere influenza season: results from the global influenza hospital surveillance network. PLoS One.

[32] Puig-Barbera J, Tormos A, Sominina A, Burtseva E, Launay O, Ciblak MA (2014). First-year results of the global influenza hospital surveillance network: 2012-2013 northern hemisphere influenza season. BMC Public Health.

[33] Mertz D, Kim TH, Johnstone J, Lam PP, Science M, Kuster SP (2013). Populations at risk for severe or complicated influenza illness: systematic review and meta-analysis. BMJ.

